# Effects of different-intensity exercise and creatine supplementation on mitochondrial biogenesis and redox status in mice

**DOI:** 10.22038/IJBMS.2022.65047.14321

**Published:** 2022-08

**Authors:** Seyhan Taskin, Taskin Celik, Seniz Demiryurek, Sibel Turedi, Abdullah Taskin

**Affiliations:** 1 Department of Physiology, Faculty of Medicine, Harran University, Sanliurfa, Turkey.; 2 Department of Physiology, Faculty of Medicine, Gaziantep University, Gaziantep, Turkey; 3 Department of Histology and Embryology, Faculty of Medicine, Harran University, Sanliurfa, Turkey; 4 Department of Nutrition and Dietetics, Faculty of Health Sciences, Harran University, Sanliurfa, Turkey

**Keywords:** Creatine, Exercise, Mitochondrial biogenesis, Nrf2, Oxidative stress

## Abstract

**Objective(s):**

Dietary supplementation combined with exercise may potentiate the beneficial effects of exercise by reducing exercise-induced oxidative stress and improving mitochondrial quality and capacity. In this study, the effects of creatine monohydrate (CrM) supplementation with low and high-intensity exercise on mitochondrial biogenesis regulators, Nrf2 anti-oxidant signaling pathway and muscle damage levels were investigated.

**Materials and Methods:**

Balb/c male mice were divided into six experimental groups: control, control+CrM, high-intensity exercise, high-intensity exercise+CrM, low-intensity exercise, and low-intensity exercise+CrM. Mice were given CrM supplementation and at the same time, low and high-intensity exercise was applied to the groups on the treadmill at 30min/5day/8week. Then, mitochondrial biogenesis marker (PGC-1α, NRF-1, TFAM), Nrf2 and HO-1 protein expressions, total oxidant-anti-oxidant status level, and histopathological changes were investigated in serum and muscle tissue.

**Results:**

Exercise intensity and CrM supplementation were found to be effective factors in mitochondrial biogenesis induction via the PGC-1α signaling pathway. Nrf2 and HO-1 protein levels increased with exercise intensity, and this result was directly related to serum oxidative stress markers. In addition, CrM supplementation was effective in reducing exercise-induced muscle damage.

**Conclusion:**

This combination induced skeletal muscle adaptations, including mitochondrial biogenesis and enhanced anti-oxidant reserves. This synergistic effect of dietary supplementation with low-intensity exercise may be valuable as a complement to treatment, especially in diseases caused by mitochondrial dysfunction.

## Introduction

Biochemical energy is needed for basic cellular processes to sustain vital activities. A mitochondrion is a unique organelle that produces ATP energy necessary for the growth and maintenance of cellular integrity (1). Mitochondria are highly influenced by the microenvironment in which cells are located (2). Before cells initiate complex processes such as adaptation to stress, proliferation, or cellular differentiation, they check whether mitochondrial metabolism is appropriate (3). It is clear that this control mechanism requires coordination between the mitochondrial and nuclear genome (1). This process, which involves the regulation of the number, size, and function of mitochondria, is referred to as mitochondrial biogenesis (4). The transcription factor PGC-1α is considered to be the primary regulator of mitochondrial biogenesis (5). Activated by phosphorylation or deacetylation, PGC-1α stimulates the transcription of nuclear respiration factor-1 and 2 (NRF-1 and NRF-2). This respiration factors transcriptionally regulates mitochondrial electron transport chain subunits encoded by the nuclear genome (6). NRF-1 also regulates transcription by binding to the promoter region of mitochondrial transcription factor A (TFAM). TFAM is the most important transcription factor regulating mitochondrial DNA transcription and replication (5). Mitochondrial biogenesis is regulated by the nuclear genome and mitochondrial transcriptional factors and is affected by oxidative stress, low temperature, calorie restriction, hypoxia, cell regeneration, differentiation, and particularly exercise (4).

Mitochondria play a central role in redox biology (7). Under physiological conditions, reactive oxygen species (ROS) are released by mitochondria as by-products of cellular respiration; mitochondria are the most important source of cellular ROS (8). Low or moderate presence of ROS plays an important role in maintaining cellular homeostasis, as they act as cellular signalers in various metabolic pathways (9). However, when they are overproduced, they cause oxidative stress and create harmful effects, which can lead to cell death. One of the most important cellular defense mechanisms against the damage caused by oxidative stress is the Nrf2 (Nuclear factor E2-related factor 2) signaling pathway (10). The initiator of the signaling pathway, Nrf2, translocates to the nucleus under stress and binds to the anti-oxidant response element gene region. Consequently, the transcription of several anti-oxidant enzymes and components, including heme oxygenase-1 (HO-1), glutathione, and superoxide dismutase, is induced (11). Exercise, which induces oxidative stress and causes the activation of anti-oxidant pathways, provides a sensitive adaptation mechanism in an organism (12). Effective ergogenic products are commonly used to maximize exercise performance, accelerate postexercise recovery, improve body condition, and maintain exercise adaptations (13). Creatine monohydrate (CrM), one of the most common dietary supplements, makes a significant contribution to cellular energy metabolism (14). Theoretically, the aim is to optimally maintain cellular bioenergetics by increasing intramuscular phosphocreatine reserves with CrM supplementation. Creatine supplementation can improve an athlete’s exercise capacity and adaptations, and these adaptations result in greater gains for the athlete in strength, muscle mass, and performance (15, 16).

Recently, the biphasic effect of oxidative stress, depending on exercise type and intensity, has been the subject of several studies (17, 18). High oxygen utilization and mitochondrial activities in the muscles during exercise can promote free radical production (19). Mitochondria, the most important source of cellular ROS, are highly sensitive to oxidative damage; damaged mitochondria can produce more ROS and less ATP (20). Strengthening the anti-oxidant defense system and improving cellular energy systems by reducing oxidative stress with the combination of exercise training and supplements are among the most important strategies. The aim of this research was to investigate the effects of low and high-intensity exercise on Nrf2 signaling pathway, oxidative stress, mitochondrial biogenesis, and muscle damage in CrM-supplemented mice.

## Materials and Methods

***Animals and exercise protocol***

The study protocol was approved by the Harran University Animal Experiments Local Ethics Committee (No: 2019/004) and conformed to the Guide for the Care and Use of Laboratory Animals. 8-10-week-old Balb/c mice (42 male) were housed in standard tranparent cages in conditions that 12 hr light:12h dark cycle with food and water *ad libitum* at 22 ± 1°C. The experimental animals were obtained from the Harran University Animal Experiment Application And Research Center. Mice were equally and randomly divided into six groups: (1) Control (C): Mice fed a standard chow diet and not exercised, (2) Low-intensity exercise (LIE): Mice fed a standard chow diet and exercised (8 m/min, 30 min with a 0° angle inclination; exercise was performed for five days per week for eight weeks), (3) High-intensity exercise (HIE): Mice fed a standard chow diet and exercised (24 m/min, 30 min with a 0° angle inclination; exercise was performed for five days per week for eight weeks), (4) Control+ CrM (C + CrM): Mice fed a standard chow diet containing CrM and not exercised, (5) Low-intensity exercise + CrM (LIE + CrM): Mice fed a standard chow diet containing CrM and exercised (8 m/min, 30 min. with a 0° angle inclination; exercise was performed for five days per week for eight weeks), (6) High-intensity exercise + CrM (HIE + CrM): Mice fed a standard chow diet containing CrM and exercised (24 m/min, 30 min with a 0° angle inclination; exercise was performed for five days per week for eight weeks). CrM was supplemented at 40 mg per kilogram of diet daily for eight weeks. The animals were acclimated to the treadmill exercise at 4 m/min for 5 min per day for 5 consecutive days before the experiment protocol (Ugo Basile, Animal Treadmill, ITALY). This exercise design was prepared by modifying earlier studies (21, 22). The running test was done at the same time every day. 

***Sample collection***

At the end of the 8 weeks (48 h post-exercise), mice were euthanized via exsanguination under deep anesthesia. Blood sample was collected from the heart and the separated serum stored at -86 °C for oxidative stress marker analyses. Skeletal muscle (*m. soleus* and* m. gastrocnemius*) tissues were excised and quickly frozen at -86 °C for western blot and mitochondrial biogenesis analyses. Different homogenization procedure was applied for each test included in our research.

***Mitochondrial biogenesis levels***

Skeletal muscle tissue PGC-1α (cat. no. MBS1601099) TFAM (cat. no. MBS2020535), NRF-1 (cat. no. MBS2704603) and NRF-2 (cat. no. MBS706836) levels were determined using commercial ELISA kit following the manufacturer’s instructions (MyBioSource, Inc.). Quality control assays assessing reproducibility the the intra- and inter-assay coefficients of variation (CVs) were less than ≤ 10%. All analyses were done with a microplate reader (Varioskan™ LUX; ThermoFisher Scientific). According to the kit instructions, skeletal muscle tissue samples were homogenized with lysis buffer. The total protein concentration in tissue was determined using the bicinchoninic acid assay method.

***Determination of oxidative stress***

Serum total oxidant status (TOS) levels was analyzed with a method developed by Erel (23). As stated in the working principle of the test, oxidants convert ferrous ions to ferric ions in the presence of o-dianisidine. The oxidation reaction that causes ferric ion formation is colored with xylenol orange compound in acidic environment. The density of the color is proportional to the level of oxidants. Hydrogen peroxide (H_2_O_2_) was used as the calibrator. The results were reported in micromole H_2_O_2_ equivalent/ L (H_2_O_2_ equiv./L). Total anti-oxidant status (TAS) levels, the other component of oxidative balance, was analyzed using the Erel method (24). Erel’s method is based on the bleaching-decolorizing of the characteristic color of stable ABTS (2,2′-azino-bis[3-ethylbenz-thiazoline-6-sulfonic acid]) radical cation by anti-oxidants. The experiments were performed by spectrophotometrically at 660 nm with Microplate Reader (ThermoFisher Scientific Varioskan™ LUX multimode microplate reader, USA). The results were reported in millimole Trolox equivalent/L. The percentage of TOS level to TAS level was regarded as the oxidative stress index (OSI) (23). The OSI value was calculated as follows: OSI= [(TOS, micromole H_2_O_2_ equivalents per liter)/(TAS, millimole Trolox equivalents per liter)×100].

***Nrf2 and HO-1 levels with western blot analysis***

Muscle tissues were lysed in RIPA Lysis Buffer (Thermo Scientific, USA), adding phosphatase and protease inhibitor cocktail (Thermo Scientific, USA). Total extracted protein concentration was determined using the bicinchoninic acid method and equal amounts of proteins (30 μg) were loaded onto 12% gradient SDS-PAGE gels and then transferred to a polyvinylidene difluoride (PVDF) membranes (Bio-rad, USA). The membranes were blocked with 5% fat-free milk in TBST buffer and incubated overnight at 4 °C with primary antibodies to Nrf2 (ab92946, 1:500, Abcam, Cambridge) and HO-1 (ab13243, 1:1000, Abcam, Cambridge). Then, the membranes were washed and incubated with secondary antibody Anti-Rabbit IgG H&L (ab205718, 1:10000, Abcam, Cambridge) at room temperature. β-actin (ab16039, 1:500, Abcam, Cambridge) was used as the loading control. Chemiluminescence substrate Super Signal West Pico (Thermo Scientific, USA) was used to visualize the immunoreactive bands. Densitometry quantification of immunoblot signals was performed using ImageJ software (National Institutes of Health, Bethesda, MD, USA) and values were normalized with loading control. All assays were performed in triplicate.

***Histopathological analysis***

At the end of the experiment period, *m. soleus* tissue samples of all groups were fixed in 10% formalin solution. 5 µm sections were taken from the paraffin blocks obtained after the routine histological tissue follow-up procedure with a semi-automatic microtome (Thermo Shandon Finesse ME+ Microtome, Runcorn, UK). Histopathological evaluation was performed by staining with Hematoxylin & Eosin (H&E) and Masson Trichrome (Sigma Aldrich, USA). In skeletal muscle tissues stained with H&E and Masson Trichrome, in ten different areas in each section; Intramuscular edema, inflammatory cell infiltration, vascular congestion, myofibril degeneration, myocyte loss, increase in the capillary and connective tissue in the intramuscular area were evaluated semiquantitatively (25, 26). All examinations were photographed using a Zeiss Axioscope II (Carl Zeiss Microscopy GmbH, Göttingen, Germany) microscope, with a Zeiss Axiocam MRc camera model attachment (Carl Zeiss MicroImaging GmbH, Göttingen, Germany) and recorded on the computer.

***Statistical analysis***

All of statistical analysis was made with SPSS 25.0 package program (SPSS Inc., Chicago, IL, USA). The sample size was determined as 42 using the G-power program (Version 3.1.9.4, Germany) taking effect size moderate (based on a similar study result), α=0.05, power (1-β)=0.80 at a confidence level of 95%. All data were checked for normality with the Kolomogorov-Smirnov test and Levene’s tests, respectively. All results regarding the mitochondrial biogenesis, oxidative stress, western blotting and histological findings were statistically analyzed using one-way ANOVA with appropriate post hoc (Tukey’s HSD test) test. Significance level was considered at *P*<0.05.

## Results

***Effect of creatine supplementation and exercise on*** ***mitochondrial biogenesis parameters***

As shown in Table 1, statistically significant differences were found between the groups in terms of PGC-1α, NRF-1 and TFAM levels (*P* ˂0.001; *P*˂0.001; *P*= 0.037, respectively); however, differences in the NRF-2 levels were not significant (*P*>0.05). CrM-supplemented exercise groups had greater PGC-1α levels (*P*<0.05) than unsupplemented exercise groups. CrM supplementation significantly increased the level of NRF-1 in all groups. The NRF-1 level increase was more prominent in the CrM-supplemented low-intensity exercise group than in the other groups (*P*<0.05). Exercise intensity and CrM supplementation were found to be effective factors in mitochondrial biogenesis induction.

***Effect of creatine supplementation and exercise on*** ***Nrf2 and HO-1 protein level***

**Figure 1:**
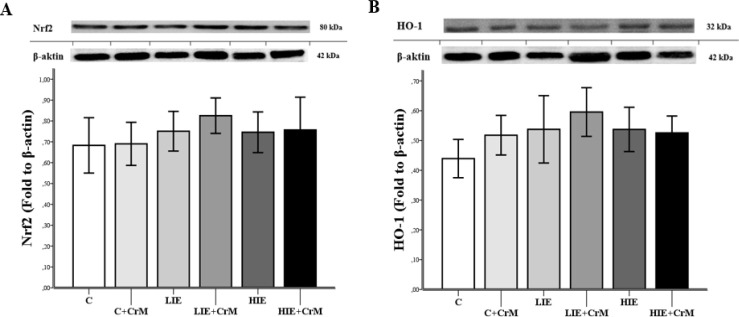
Protein expression of Nrf2 (A) and HO-1 (B) in muscle tissue was examined with western blotting. Protein level of Nrf2 and HO-1 normalized to β-actin. Values are expressed as mean ± standard deviation of three independent experiments, n=7 per group

To investigate the levels of muscle tissue Nrf2 and HO-1 protein expression, we assessed them using quantitative western blotting (n = 7/each group). After eight weeks of intensity exercise training, there was an increase in Nrf2 protein levels in the exercise groups that received CrM, but no statistically significant difference was observed (*P*> 0.05). The highest Nrf2 protein levels were found in the CrM-supplemented low-intensity exercise group (Figure 1). Figure 1 shows the effect of the exercise-induced HO-1 modalities ort he eight-week exercise with CrM supplementation. No significant difference in protein levels was observed for HO-1 in all groups. The HO-1 level in the CrM supplementation and exercise groups increased compared to the control group, but this increase was not statistically significant (*P*>0.05). The highest HO-1 protein levels were found in the CrM-supplemented low-intensity exercise group.

***Effect of creatine supplementation and exercise on markers of oxidative stress***

**Figure 2:**
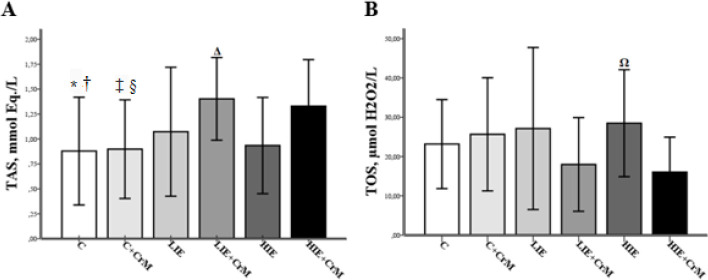
Total anti-oxidant status level (A) and total oxidant status level (B) of experimental groups. Mean ± standard deviation. There is a statistical difference between the following groups (*P*˂0.05); *: C vs LIE+CrM, †: C vs HIE+CrM, ‡: C+CrM vs LIE+CrM, §: C+CrM vs HIE+CrM, Δ: LIE+CrM vs HIE, Ω: HIE vs HIE+CrM

As shown in Table 2, statistically significant differences were found between the groups in TAS, TOS, and OSI levels (*P*=0.001, *P*=0.009, *P*˂0.001, respectively). High-intensity exercise caused an increase in oxidative stress. A higher anti-oxidant capacity was found in the exercise groups that received creatine supplementation (Figure 2). In the HIE + CrM and LIE + CrM groups, CrM supplementation tolerated the formation of exercise-induced ROS, resulting in lower TOS values. The lowest TOS levels were found in the LIE+CrM group (Figure 2).

***Histopathological evaluations of skeletal muscle***

**Figure 3:**
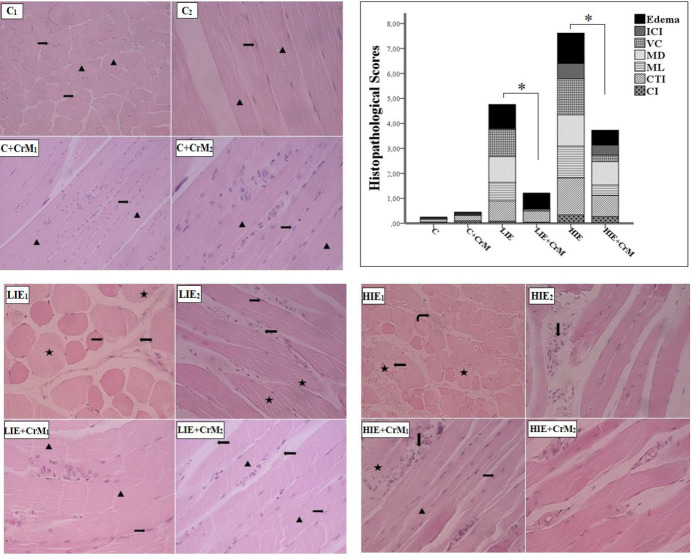
Photomicrography of transverse and longitudinal sections taken from m.soleus. (H&E, 20X, 40X)

C1: Control (Transverse); C2: Control (Longitudinal); C+CrM1-2: Control+CrM (20X-40X Longitudinal); LIE1: Low Intensity Exercise (Transverse); LIE2: Low Intensity Exercise (Longitudinal); LIE+CrM1: Low Intensity Exercise+CrM (Transverse); LIE+CrM2: Low Intensity Exercise+CrM (Longitudinal); HIE1: High Intensity Exercise (Transverse); HIE2: High Intensity Exercise (Longitudinal); HIE+CrM1-2: High Intensity Exercise+CrM (20X-40X Longitudinal) *: *P*˂0.05

**Figure 4:**
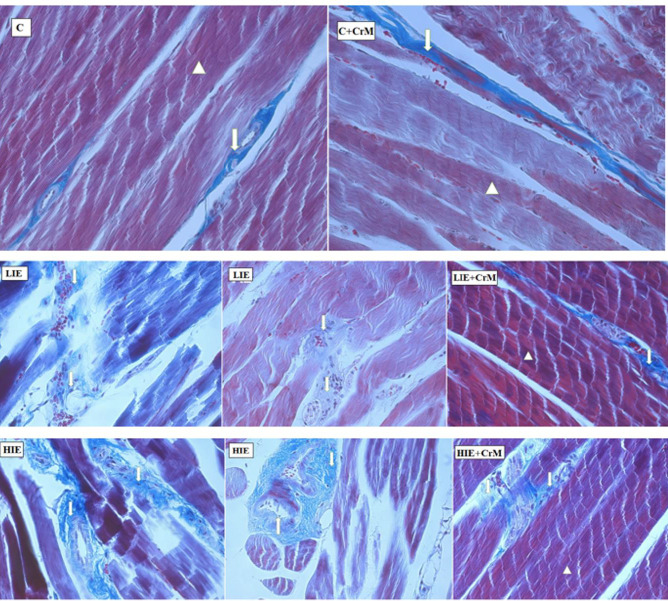
Photomicrography of longitudinal sections taken from m.soleus. (Masson Trichrome staining, 20X, 40X). C: Control; C+CrM: Control+CrM; LIE: Low Intensity Exercise; LIE+CrM: Low Intensity Exercise+CrM; HIE: High Intensity Exercise; HIE+CrM: High Intensity Exercise+CrM. Myofibril bundle (arrowhead), intramuscular connective tissue increase (down arrow)

In the longitudinal and transverse sections of the C and C + CrM groups, skeletal muscle [*m. soleus*] tissue samples were observed to have normal morphology, and no pathology was found. A small amount of intramuscular edema and increased connective tissue was observed in the LIE group, as well as disorganization of muscle fiber bundles. In the sections of the HIE group, vascular congestion, intramuscular edema, disorganization and degeneration of myofibril bundles, and an increase in the capillary and connective tissue in the intramuscular area were observed. In the LIE + CrM group, although a small amount of intramuscular edema and connective tissue was observed, the borders of the myofibril bundles and the core structure were found to have normal morphological features. In the longitudinal sections of the HIE + CrM group, it was observed that vascular congestion and degeneration of muscle fiber bundles decreased; intramuscular edema was not observed, but morphological damage to the skeletal muscle continued to some extent as a result of high-intensity exercise (Figure 3 [H&E staining]; Figure 4 [Masson trichrome staining]). Based on parameter analysis, there was a statistically significant increase in skeletal muscle score values in the LIE and HIE groups compared to control group (*P*<0.05). Moreover, when the LIE + CrM group was compared with the LIE group, a significant decrease was observed, especially in vascular congestion and myofibril degeneration (*P*<0.05). When the HIE + CrM group was compared with the HIE group, it was observed that vascular congestion decreased statistically, even though all parameters were decreased (*P*<0.05).

## Discussion

This study revealed the protective effect of CrM against exercise-induced oxidative stress and discussed the underlying molecular mechanisms through mitochondrial biogenesis adaptations and anti-oxidant defense systems. Histological analysis results showed that CrM supplementation can effectively protect muscle tissue against damage caused by contractile activity by providing cellular energy continuity during exercise. In particular, it was confirmed that exercise can significantly activate mitochondrial biogenesis, and it was revealed for the first time that CrM supplementation further potentiated this effect with exercise. CrM supplementation protected against oxidative stress by increasing Nrf2 and HO-1 protein expression levels and activating mitochondrial biogenesis.

Physical activity and exercise are often recommended to prevent chronic diseases, treat diseases, shorten the recovery period, and maintain a healthy state. Exercise causes skeletal muscle adaptations throughout the body that can affect metabolism and functions (27). However, increased metabolic activities lead to changes in redox balance and an increase in ROS production. Exercise-induced ROS serves as a signaling molecule and forms the basis of exercise adaptation mechanism (28). However, persistently high levels of ROS during repetitive or prolonged muscle contractions may cause shifts in the redox balance towards oxidative stress. Prolonged exercise can cause structural damage and contractile dysfunction in skeletal muscle (29); therefore, the optimum amount of ROS that has beneficial effects in an organism is still unclear.

Increasing evidence shows that medicinal products with ROS-scavenging effects and supplements can prevent exercise-induced oxidative stress and reduce muscle damage (17, 30). Hence, the idea of ​​using supplements along with exercise has been frequently investigated. CrM, an ergogenic effective product frequently consumed by athletes, has been stated to be effective in reducing muscle damage after exercise and has been shown to increase the activity of anti-oxidant enzymes (31, 32)^.^ However, there are conflicting results regarding the anti-oxidant effect of CrM supplementation (33, 34). In our previous study, we demonstrated that CrM supplementation has a synergistic effect with exercise and is effective in maintaining thiol-disulfide homeostasis in organisms (35). This present study revealed that CrM supplementation was effective in reducing oxidative stress associated with exercise intensity. It was found that CrM supplementation, especially with low-intensity exercise, increased anti-oxidant defense more effectively. Increasing intramuscular phosphocreatine stores with CrM supplementation is thought to mediate the formation of this effect by providing continuity in cellular energy systems during exercise.

In the long term, the changing redox state with the continuation of contractile activity at high intensity predisposes to muscle damage (36). The results of the histopathological analysis performed in this study showed that the exercise caused muscle damage in proportion to the intensity. Increased exercise intensity led to disorganization and degeneration of myofibrillar bundles. However, it was observed that the muscle damage after exercise decreased in the groups receiving CrM supplementation. CrM consumption, especially with high-intensity exercise, was more effective in reducing muscle damage caused by contractile activity. Previous studies have reported that CrM supplementation with regular exercise can cause cell membrane stabilization and improved cellular energy capacity (32, 37). It is thought that CrM is effective in the restoration of damaged muscle morphology through these effects.

Body systems are exposed to several stress factors during exercise. This process stimulates multiple signaling pathways that regulate adaptive responses, including gene expressions (38). Nrf2, a component of adaptive responses to exercise training, is an important transcription factor that regulates the expression of anti-oxidant proteins and enhances the anti-oxidant defense system against oxidative damage (10). Exercise can modulate Nrf2 protein expression and signaling—it has been reported that acute exercise causes an increase in the expression of Nrf2 proteins (39). However, there are different results in studies showing the effects of regular exercise (or chronic exercise) on Nrf2 protein expression (40, 41). Exercise applied at optimum levels with its duration and intensity can cause significant redox profile changes in skeletal muscle, resulting in a more resistant capacity against ROS (42). Adaptations that occur in response to regular exercise affect aerobic capacity. Increased exercise intensity increases oxidative stress, but the increased adaptive capacity of muscles can overcome oxidative stress (43, 44). In our study, it was found that CrM supplementation applied together with low-intensity exercise improved the adaptive capacity of the muscles and caused an increase in Nrf2 and HO-1 protein expression. This is consistent with the findings of serum oxidative stress. The presence of higher TAS levels and lower TOS levels, especially in the low-intensity exercise group receiving CrM supplementation, is an indicator of an enhanced endogenous anti-oxidant defense system. CrM supplementation can be considered as one of the strategies that limit the possible amount of ROS that can be formed during exercise by increasing the capacity to adapt to exercise. The adaptive response of the anti-oxidant defense against the formation of ROS suggests the existence of new compensation processes.

One of the critical mediators of the adaptive response to exercise training is mitochondrial biogenesis (45). It is known that exercise is a powerful stimulant that can cause functional and structural changes in mitochondria and improve the quantity and quality of the organelle network (46). Increased mitochondrial biogenesis improves oxidative phosphorylation and respiratory capacity per mitochondria (47). Exercise activates multiple signaling pathways that converge to initiate mitochondrial biogenesis (48). Acute muscle contraction, associated with Ca^2+^ movements and ATP/ADP conversions, causes an increase in ROS production, especially from mitochondria and other cellular reactions (49). This activates PGC-1α, which is considered a central regulator of mitochondrial biogenesis (46). Studies among human and mouse/rat have reported that PGC-1α levels increase after different exercises, such as cycling, swimming, and running (50–52). It has been found that in response to the increase in ADP sensitivity during chronic exercise, the direction of energy metabolism is maintained by oxidative phosphorylation reactions rather than anaerobic glycolysis (53). One of the most prominent responses to chronic exercise training has been reported to be the release of less ROS compared to acute exercise. This has been attributed to a more tightly regulated maximal capacity regulation of mitochondrial biogenesis and oxidative phosphorylation reactions in trained muscles (53). 

This study confirmed that exercise can significantly activate mitochondrial biogenesis, and for the first time, CrM supplementation was found to further potentiate this effect with exercise. In our study, the increase in PGC-1α, NFR-1, and TFAM levels, especially in the groups that received CrM supplementation with exercise, led us to conclude that CrM induced mitochondrial biogenesis. In particular, this effect became more pronounced with low-intensity exercise. It is critical to ensure energy continuity, especially in active muscles, with the increased metabolic rate during exercise. It is thought that storage phosphocreatine, which is increased in muscle with creatine supplementation (16), improves cellular energy systems by facilitating the transition process to oxidative phosphorylation during exercise. In relation to its intensity, exercise and CrM supplementation resulted in improvements in mitochondrial capacity and function. This information may be promising for many mitochondria-related diseases.

**Table 1:** Effect of creatine supplementation and exercise on mitochondrial biogenesis markers

	**C**	**C+CrM**	**LIE**	**LIE+CrM**	**HIE**	**HIE+CrM**	***p***
**NRF-1, pg/mg protein**	147.3±18.5^*^^, †^	181.2±20.2^‡^^, §^	169.3±38.6 ^||^^, ¶^	361.3±89.6^Δ,Σ^	166.5±24.8^Ω^	267.6±51.0	˂0.001
**NRF-2, pg/mg protein**	910.8±158.7	894.6±512.0	791.1±298.6	858.5±305.9	1180.3±290.3	1190.1±628.2	0.288
**PGC-1α, ng/mg protein**	2.84±0.2^†^	2,72±0.2^‡^^,§^	3.07±0.4^#^	3.38±0.4^Δ^	2.38±0.2^Ω^	3.58±0.5	˂0.001
**TFAM, ng/mg protein**	424.3±119.0	398.0±112.9	378.1±77.7^¶^	515.0±134.3	536.9±176.6	579.2±158.2	0.037

Mean ± standard deviation. There is a statistical difference between the following groups (*P*˂0.05); *: C vs LIE+CrM, †: C vs HIE+CrM, ‡: C+CrM vs LIE+CrM, §: C+CrM vs HIE+CrM, ||: LIE vs LIE+CrM, ¶: LIE vs HIE+CrM, #: LIE vs HIE, Δ: LIE+CrM vs HIE, Σ: LIE+CrM vs HIE+CrM, Ω: HIE vs HIE+CrM. Peroxisome proliferator-activated receptor gamma coactivator 1 alpha (PGC-1α), Nuclear respiration factor-1 (NRF-1), Nuclear respiration factor-2 (NRF-2), Mitochondrial transcription factor A (TFAM)

C: Control, C+Crm: Control+Creatine monohydrate, LIE: Low Intensity Exercise, LIE+CrM: Low Intensity Exercise+Creatine monohydrate, HIE: High Intensity Exercise, HIE+CrM: High Intensity Exercise+Creatine monohydrate

C: Control, C+CrM: Control+Creatine monohydrate, LIE: Low Intensity Exercise, LIE+CrM: Low Intensity Exercise+Creatine monohydrate, HIE: High Intensity Exercise, HIE+CrM: High Intensity Exercise+Creatine monohydrate

**Table 2:** Oxidative stress parameters after eight weeks of exercise training with creatine monohydrate supplementation

	**C**	**C+CrM**	**LIE**	**LIE+CrM**	**HIE**	**HIE+CrM**	***p***
**TAS, mmol Eq./L**	0.87 ± 0.27 ^*, †^	0.89 ± 0.24 ^‡^^, §^	1.07 ± 0.32	1.40 ± 0.20 ^Δ^	0.93 ± 0.24	1.32 ± 0.23	0.001
**TOS, µmol H**_2_ **O**_2_ **/L**	23.17 ± 5.65	25.65 ± 7.20	27.13 ± 10.30	17.98 ± 5.95	28.48 ± 6.79 ^Ω^	16.04 ± 4.43	0.009
**OSI, AU**	2.72 ± 0.63 ^*, †^	2.90 ± 0.63 ^‡^^, §^	2.58 ± 0.87 ^||, ¶^	1.32 ± 0.54 ^Δ^	3.20 ± 0.96 ^Ω^	1.26 ± 0.48	˂0.001

Mean ± standard deviation. There is a statistical difference between the following groups (*P*˂0.05); *: C vs LIE+CrM, †: C vs HIE+CrM, ‡: C+CrM vs LIE+CrM, §: C+CrM vs HIE+CrM, ||: LIE vs LIE+CrM, ¶: LIE vs HIE+CrM, Δ: LIE+CrM vs HIE, Ω: HIE vs HIE+CrM

C: Control, C+Crm: Control+Creatine monohydrate, LIE: Low Intensity Exercise, LIE+CrM: Low Intensity Exercise+Creatine monohydrate, HIE: High Intensity Exercise, HIE+CrM: High Intensity Exercise+Creatine monohydrate

C: Control, C+CrM: Control+Creatine monohydrate, LIE: Low Intensity Exercise, LIE+CrM: Low Intensity Exercise+Creatine monohydrate, HIE: High Intensity Exercise, HIE+CrM: High Intensity Exercise+Creatine monohydrate

ICI: Inflammatory cell infiltration, VC: Vascular congestion, MD: Myofibrillar degeneration, ML: Myocyte loss, CTI: Connective tissue increase, CI: Capillary increase. Myofibril bundle (arrowhead), myocyte (right arrow), myofibril degeneration (star), intramuscular edema (left arrow), vascular congestion (bent arrow), connective tissue enlargement (down arrow)

## Conclusion

Controlling the amount of ROS produced with the intensity of exercise requires a delicate balance. Maintaining this balance is possible with supplements and optimal exercise duration and intensity. CrM supplementation was effective in tolerating exercise-induced ROS generation. CrM supplementation with exercise was induced skeletal muscle adaptations, including mitochondrial biogenesis and enhanced anti-oxidant reserves. Our findings demonstrated that low-intensity exercise and CrM supplementation can improve mitochondrial biogenesis, ensure the continuity of oxidant-anti-oxidant homeostasis, and optimize the transformation of this dynamic balance.

## Authors’ Contributions

ST Methodology, conceptualization, investigation, formal analysis, writing - original draft, writing – review and editing. HC Methodology, conceptualization, investigation, formal analysis, writing - review & editing. SD Methodology, conceptualization, writing - review & editing. ST Investigation, Formal analysis, writing - review & editing. AT Investigation, formal analysis, writing - review & editing. All authors read and approved the final version of the manuscript.

## Financial Support

This work was supported by the Harran University Scientific Research Project Unit (Grant number #19325).

## Conflicts of Interest

The authors have no competing interests to declare that are relevant to the content of this article.
